# White matter integrity in young medication-naïve bipolar II depressed adults

**DOI:** 10.1038/s41598-021-81355-9

**Published:** 2021-01-19

**Authors:** Arthur Dun Ping Mak, Owen Ngo Wang Leung, Idy Wing Yi Chou, Sheila Lok Yiu Wong, Winnie Chiu-wing Chu, David Yeung, Suzanne Ho-wai So, Suk Ling Ma, Linda Chiu Wah Lam, Chi Ming Leung, Sing Lee

**Affiliations:** 1 Department of Psychiatry, G/F Multicentre, Tai Po Hospital, The Chinese University of Hong Kong, Tai Po, Hong Kong, SAR China; 2 Department of Imaging and Interventional Radiology, The Chinese University of Hong Kong, Hong Kong, SAR China; 3 Department of Clinical Oncology, Prince of Wales Hospital, Hong Kong, SAR China; 4 Department of Psychology, The Chinese University of Hong Kong, Hong Kong, SAR China

**Keywords:** Bipolar disorder, Depression

## Abstract

It is unknown if young medication-naïve bipolar II (BPII) depressed patients have increased white matter (WM) disruptions. 27 each of young (average 23 years) and treatment-naïve BPII depressed, unipolar depressed (UD) patients and age–sex–education matched healthy controls (HC) underwent 3 T MRIs with diffusion tensor imaging. Diagnostic ratings included Structured Clinical Interview for DSM Disorders (SCID), Montgomery-Åsberg Depression Rating Scale (MADRS), Young Mania Rating Scale (YMRS) and Hamilton Anxiety Rating Scale (HAM-A). Patients were clinically depressed (MADRS-BPII: 26.15 [SD9.25], UD: 25.56 [5.24], p = 0.86). Compared to UD, BPII had increased family bipolarity (BPII 13.6% vs UD 2.5%, p = 0.01, φc = 0.28), hypomanic symptoms (YMRS-BPII: 4.22 [4.24], UD: 1.33 [2], p = 0.02, d = 0.87), lifetime number of depressive episodes (BPII: 2.37 [1.23], UD: 1.44 [0.75], p = 0.02, d = 0.91), lifetime and current-year number of episodes (lifetime BPII: 50.85 [95.47], UD: 1.7 [1.03]; current-year BPII: 9.93 [16.29], UD: 1.11 [0.32], ps = 0.04, ds = 0.73–0.77) and longer illness duration (BPII: 4.96 years [3.96], UD: 2.99 [3.33], p = 0.15, d = 0.54). BPII showed no increased WM disruptions vs UD or HC in any of the 15 a priori WM tracts. UD had lower right superior longitudinal fasciculus (SLF) (temporal) axial diffusivity (AD) (1.14 vs 1.17 (BPII), 1.16 (HC); F = 6.93, *95% CI of* ${F}_{B}$: 0.00073, 5.22, ηp^2^ = 0.15). Principal component analysis followed by exploratory linear discriminant analysis showed that increased R-SLF (temporal) AD, YMRS and family bipolarity distinguished BPII from UD (81.5% sensitivity, 85.2% specificity) independent of episode number and frequency. Young, medication-naïve adults with BPII depression did not show the WM disruptions distinguishing more chronically ill BP patients from UD. These WM disruptions may therefore be partly attributable to illness chronicity. Longitudinal studies should examine the trajectory of WM changes in BPII and UD and predictive validity of these baseline clinical and imaging parameters.

## Introduction

Bipolar II disorder (BPII) is the commonest subtype of bipolar disorders^1^. Depression dominates the course of BPII and is the commonest mode of clinical presentation of the disorder. This phenomenological overlap with unipolar depression (UD) belies important clinical differences—poorer response to antidepressants, younger onset, higher recurrence, atypical features, cognitive impairment and increased suicide rates compared with UD^2–5^. Understanding the neurobiological differences between BPII depression and UD should yield important insights into the core etiological mechanisms differentiating between bipolar and unipolar illnesses and guide therapeutic development.

Diffusion tensor imaging (DTI) studies on bipolar disorders have found white matter (WM) connectivity disruption, consistent with a model of emotional dysregulation, in the fronto-limbic circuitry (cingulum, uncinate fasciculus), inter-hemispheric circuitry (corpus callosum), the fronto-parieto-temporal long associative fibers, and frontal and temporal regions, compared to healthy subjects^6–9^, with more widespread disruptions compared to UD^7,10–12^. There has been a paucity of direct comparisons between UD and BPII, and the findings above were mostly based on bipolar I (BPI) samples. Findings from the few studies pertaining to BPII were also less consistent. One study reported increased fiber alterations in temporal and inferior prefrontal region in BPII^13^. while others found sparing of WM disruption in uncinate fasciculus^14^ and the corpus callosum^9^. It is also uncertain whether the contradicting findings were attributable to heterogeneity in illness chronicity (7.3–18.8 years)^7–10,13^. In fact, while WM disruptions has been proposed as an endophenotypic/trait marker of bipolarity^15^, oxidative stress from longer illnesses with more illness episodes may result in increased myelin disruption in bipolar disorders^16^. Heterogeneity in illness chronicity in the samples may therefore potentially contribute to the inconsistent findings. In addition, exposure to medications such as lithium^17^, antipsychotics^17^, anticonvulsants^18^, antidepressants^19^ in Bipolar Disorder (BD) has been known to result in changes in WM integrity that may further add heterogeneity to the results. This may be especially relevant to UD vs BPII comparisons in view of the considerable difference in medications prescribed for these two conditions. We therefore set out to examine WM abnormalities in treatment-naïve BPII subjects relatively close to onset, in comparison with UD and healthy controls (HC). DTI derived indices of WM integrity (fractional anisotropy [FA], mean diffusivity [MD], radial diffusivity [RD], and axial diffusivity [AD]) are obtained for 15 a priori, well-characterized white matter tracts implicated in BD^18,20^. The between-group differences are examined with a multi-variate statistical model to differentiate BPII from UD.

## Results

### Demographics

Twenty-seven each of BPII, UD and HC subjects were included in the analyses. There was no significant difference of age (F[2,78] = 0.79, *p* = 0.46), gender (X^2^[2, N = 81] = 1.5, *p* = 0.47), and years of education (X^2^[4, N = 81] = 5.12, *p* = 0.28) amongst the three groups (Supplementary Table S1).

### Clinical characteristics in BPII and UD patients

All patients were currently in a major depressive episode (MDE), as defined in the text revision of the fourth edition of the Diagnostic and Statistical Manual of Mental Disorders (DSM-IV-TR), of at least moderate severity. UD and BPII patients had no significant between-group difference in Montgomery–Åsberg Depression Rating Scale (MADRS) scores (t[52] = 0.29, p = 0.86) or functional impairment (Short-form-36 health survey physical component summary [SF-36 PCS] t[52] = − 0.2, p = 0.89; mental component summary [MCS] t[52] = 0.68, p = 0.73), but mean current Young Mania Rating Scale (YMRS) score (t[52] = 3.2, *p* = 0.02, Cohen’s d = 0.87) was significantly higher in BPII. The bipolar patients have had on average 5 years since first depressive onset, numerically but not significantly longer than UD (t[52] = 1.97, p = 0.15). BPII patients had larger lifetime number of depressive episodes (t[52] = 2.41, *p* = 0.02, Cohen’s d = 0.91), number of episodes in the past year (t[52] = 2.81, *p* = 0.04, Cohen’s d = 0.77), lifetime number of episodes (t[52] = 2.64, *p* = 0.04, Cohen’s d = 0.73), family history of hypomania (HM)(X^2^[1, N = 81] = 6.48, *p* = 0.01, *φc* = 0.28), and bipolarity Index (t[52] = 6.54, *p* < 0.001, Cohen’s d = 1.78) than UD. HC had significantly higher estimated IQ score than BPII (*p* = 0.03) and UD (*p* = 0.01) patients (F[2,77] = 5.07, p = 0.009, ηp^2^ = 0.12) (Supplementary Table S1).

### Group differences of white matter measures

#### TRACULA

**Figure 1:**
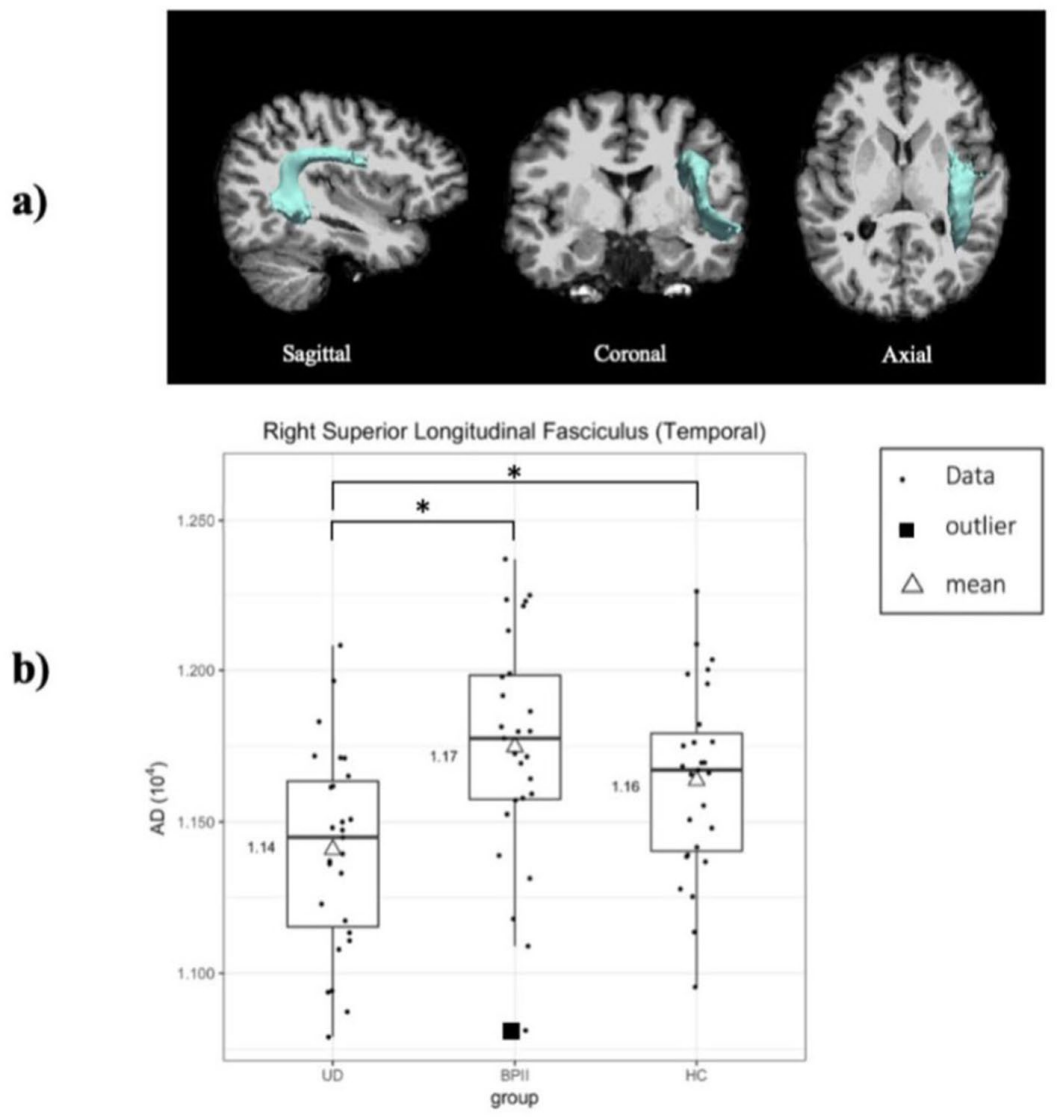
(**a**) Right superior longitudinal fasciculus (temporal) overlaid on the T1-weighted MR images (in native space) of a representative subject. Pathway was retrieved from the 4D volume provided by TRACULA. (**b**) Box plots of axial diffusivity at the right superior longitudinal fasciculus (temporal).

Bootstrapped ANOVA was done on each DTI measure at each tract with 1000 iterations and the bootstrapped ${F}_{B}$ were extracted. Significant difference was found only for AD at the right superior longitudinal fasciculus (SLF) (temporal) (1.14 (UD), 1.17 (BPII), 1.16 (HC); F[2,78] = 6.93, *95% confidence interval (CI) of* ${F}_{B}$: 0.00073, 5.22; ηp^2^ = 0.15). No significant group differences of DTI measures were found in any of the remaining tracts (*ps* > 0.05; observed *F* fell within the 95% CIs of ${F}_{B}$). Post-hoc t-tests indicated significantly lower AD at the right SLF (temporal) in UD compared to BPII and HC (t statistic not within 95% CIs of bootstrapped t statistic, Cohen’s d vs BPII = 1; Cohen’s d vs HC = 0.67), but no significant difference between BPII and HC (Table 1 and Fig. 1).

**Table 1:** DTI measures at right superior longitudinal fasciculus and ANOVA results.

Measure	UD (N = 27)	BPII (N = 27)	HC (N = 27)	DOF	F	95% CI of ${\mathrm{F}}_{\mathrm{B}}$	ηp^2^					
FA	0.48 (0.02)^a^	0.48 (0.02)^a^	0.48 (0.02)^a^	2, 78	0.78	0.00075, 40.89	0.02					
AD	10.14 (0.03)^a,b^	10.17 (0.03)^a,b^	10.16 (0.03)^a,b^	2, 78	60.93^c^	0.00073, 50.22	0.15					
RD	0.52 (0.02)^a,b^	0.53 (0.02)^a,b^	0.53 (0.03)^a,b^	2, 78	0.68	0.00115, 40.94	0.02					
MD	0.73 (0.02)^a,b^	0.75 (0.02)^a,b^	0.74 (0.02)^a,b^	2, 78	30.65	0.00152, 40.84	0.09					

Post-hoc												
	UD vs. BPII				UD vs. HC				BPII vs. HC			
	DOF	t	95% CI of ${t}_{B}$	Cohen's D	DOF	t	95% CI of ${t}_{B}$	Cohen's D	DOF	t	95% CI of ${t}_{B}$	Cohen's d
AD	52	3.50^c^	− 1.93, 2.03	1	52	2.62^c^	− 2.06, 2.12	0.67	52	− 1.17	− 1.92, 1.93	0.33

*CI* confidence interval, ${F}_{B}$ bootstrapped F statistics, *DOF* degrees of freedom, ${t}_{B}$ bootstrapped t statistic, *UD* Unipolar Depression, *BPII* Bipolar II Disorder, *HC* healthy control, *FA* fractional anisotropy, *AD* axial diffusivity, *RD* radial diffusivity, *MD* mean diffusivity.

^a^Mean (SD).

^b^Multiply by 10^–3^.

^c^Statistically significant, where the observed statistics was not within the 95% CIs of the bootstrapped statistics.

To explore the effect of age on the DTI measures in each diagnostic group, group-by-age ANOVA with bootstrapping was conducted (Supplementary Table S2). Results revealed significant interaction between group and age on MD at the right inferior longitudinal fasciculus (ILF) (7.73 × 10^−4^ (UD), 7.82 × 10^−4^ (BPII), 7.74 × 10^−4^ (HC); *F* = 5.28, 95% *CI of F*_*B*_ 0.001, 5.02). Post-hoc one-way ANOVA showed significant effect of age in BPII only (*F* = 5.28, *95% CI of F*_*B*_ 0.001, 5.02), but not UD and HC (observed *F* fell within *95% CI* of bootstrapped *F*_*B*_).

#### TBSS

Permutation tests with threshold-free cluster enhancement (TFCE) revealed no significant between-group voxel-wise difference of FA, AD and RD (*ps* > 0.05), adjusted for age, sex and education level.

### Correlations between TRACULA and clinical data

No significant correlation between clinical and DTI variables were found when data of both patient groups were combined (observed *r* fell within the 95% CIs of ${r}_{B}$). Correlation was also examined for each patient group separately. In the UD group, AD at right superior longitudinal fasciculus (SLF; temporal) correlated negatively with family history of major depressive disorder (MDD; *r* = − 0.307, 95% *CI* of ${r}_{B}$ − 0.196, 0.204). In BPII, AD of right SLF (temporal) positively correlated with family history of MDD (*r* = 0.203, 95% *CI* of ${r}_{B}$ − 0.169, 0.136), and negatively with Bipolarity Index (*r* = − 0.121, 95% *CI* of ${r}_{B}$ − 0.109,0.585). Correlations between AD at right SLF and other clinical variables were not significant (*ps* > 0.05; observed *r* fell within the 95% CIs of ${r}_{B}$) (Table 2).

**Table 2:** Correlations between clinical variables and AD at the right SLF (temporal).

Clinical variable	UD + BPII (N = 54)		UD only (N = 27)		BPII only (N = 27)	
	*r*	*95% CI of* ${r}_{B}$	*r*	*95% CI of* ${r}_{B}$	*r*	*95% CI of* ${r}_{B}$
MADRS	− 0.027	− 0.315, 0.207	− 0.160	− 0.400, 0.373	0.004	− 0.400, 0.394
YMRS	0.351	0.135, 0.522	0.049	0.039, 0.633	0.292	− 0.109, 0.503
HAM-A	− 0.309	− 0.483, 0.066	− 0.114	− 0.572, 0.164	− 0.515	− 0.561, 0.171
Year since depressive onset	0.159	− 0.074, 0.384	0.011	− 0.169, 0.508	0.080	− 0.194, 0.456
Total number of MDE	0.204	− 0.084, 0.448	− 0.046	− 0.209, 0.539	0.070	− 0.213, 0.547
Number of episodes in the past year	0.285	− 0.036, 0.475	0.055	− 0.123, 0.552	0.200	− 0.126, 0.565
Lifetime total number of episodes	0.215	0.043, 0.407	− 0.106	− 0.077, 0.517	0.102	− 0.060, 0.496
Bipolarity Index	0.252	− 0.007, 0.477	0.040	− 0.097, 0.518	− 0.121*	− 0.109, 0.585
Lifetime number of comorbid disorders	0.113	− 0.105, 0.352	0.286	− 0.205, 0.496	0.134	− 0.230, 0.418
Lifetime number of anxiety disorders	0.105	− 0.163, 0.350	0.281	− 0.285, 0.500	0.108	− 0.289, 0.390
Family history of MDD	− 0.007	− 0.128, .113	− 0.307*	− 0.196, .204	.203*	− 0.169, .136
Family history of HM	.200	− 0.045, .417	− 0.085	− 0.171, .580	.093	− 0.341, .325
SF-36 PCS	0.011	− 0.238, 0.287	− 0.043	− 0.370, 0.414	0.081	− 0.358, 0.430
SF-36 MCS	0.125	− 0.140, 0.379	0.058	− 0.275, 0.485	0.118	− 0.281, 0.455

*MADRS* Montgomery–Åsberg Depression Rating Scale, *YMRS* Young Mania Rating Scale, *HAM-A* Hamilton Anxiety Rating Scale, *MDE* Major Depressive Episode, *HME* Hypomanic Episode, *SF-36* 36-Item Short Form Survey, *PCS* Physical component summary, *MCS* Mental component summary, *CI* confidence interval, ${r}_{B}$ Bootstrapped Pearson correlation coefficient.

*Statistically significant, where the observed statistics was not within the 95% CIs of the bootstrapped statistics.

### Predictive models

**Figure 2:**
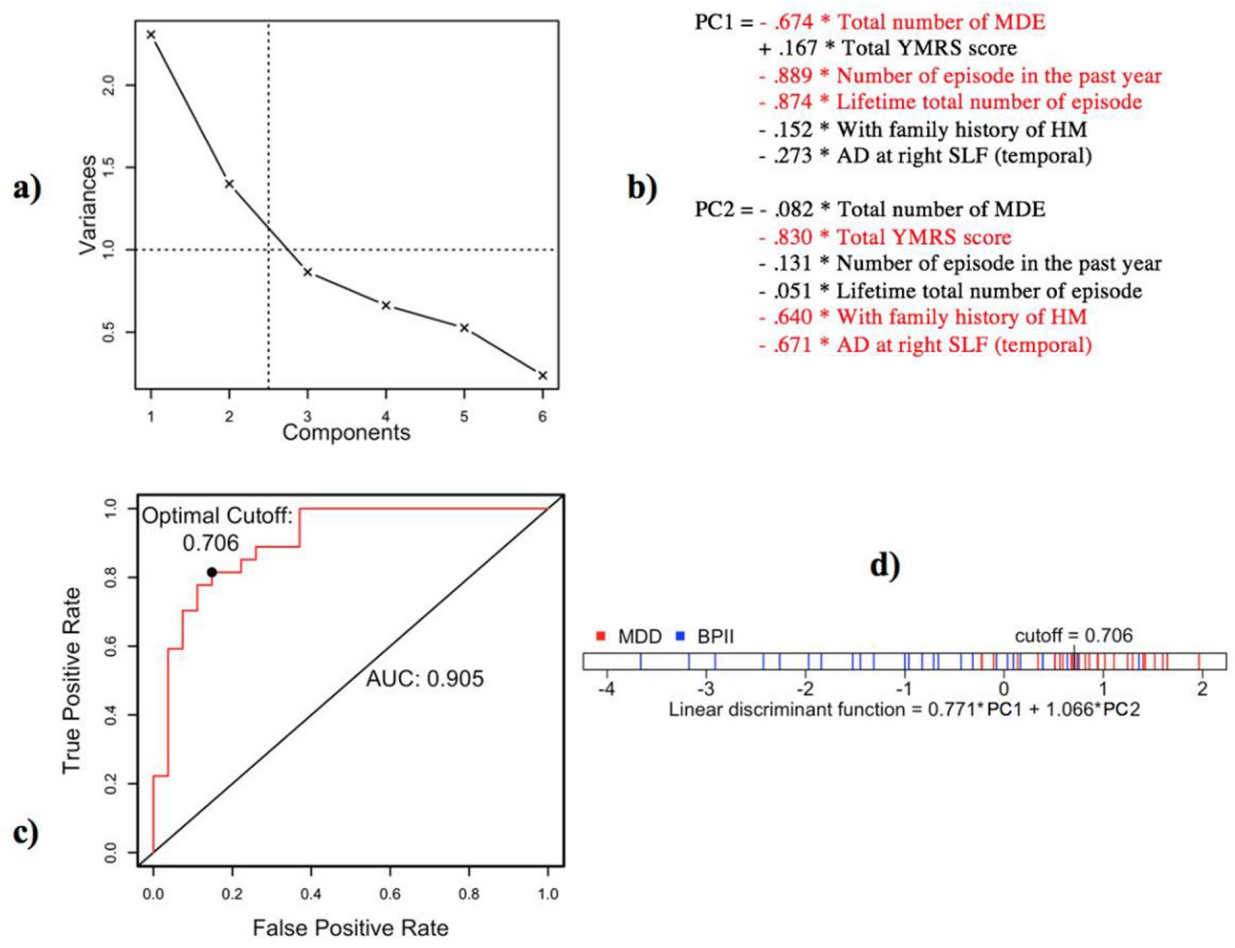
(**a**) Scree plots showing the amount of variance explained by each component in the principal component analysis. (**b**) Principal component analysis loadings. (**c**) Receiver operating curve of the function. (**d**) Linear Discriminant Function.

Principal component analysis (PCA) results are illustrated in Fig. 2a,b. To understand the relationship between imaging and clinical variables, we first included five clinical and one DTI variables with significant differences between UD and BPII in the PCA: (1) total number of Major Depressive Episodes, (2) number of episodes in the past year, (3) total YMRS score, (4) lifetime total number of episodes, (5) family history of HM, and (6) AD at right SLF (temporal). Bipolarity Index was not included in the analysis as its inherent multi-dimensional nature would make it difficult to interpret in the multivariate model.

According to the scree plot, two components were found to account for 61.9% of variance in our data (Fig. 2a).

#### Component 1 (PC1): Episodic recurrence

Component 1 included three variables of medium-to-high negative loadings, namely lifetime total number of MDE, lifetime total number of episodes and the number of episodes in the past year.

#### Component 2 (PC2): WM integrity and family history/severity of hypomania

Component 2 included three medium-to-high negative loadings for right SLF (temporal) axial diffusivity, current YMRS score and family history of bipolar disorder.

These two components were then entered into linear discriminant analysis (LDA, which resulted in a linear discriminant function: 0.771 × PC1 + 1.066 × PC2, with 81.5% sensitivity and 85.2% specificity (Fig. 2c,d). Value above the cut-off at 0.706 indicated UD, whereas value below the cut-off indicated BPII.

A second PCA was then run with only clinical variables ((1) to (5)) to clarify the effect of inclusion of right SLF (temporal) AD in the model (Supplementary Figure S1). Results revealed a grossly similar component structure, where two components of episodic recurrence and family history/hypomania severity explained 67.6% of variance in the data, and the LDA classified patient groups with 69.2% sensitivity and 92.6% specificity.

## Discussion

We did not find evidence of increased white matter disruption in our sample of young and treatment-naïve depressed bipolar II patients, in comparison with age/sex/education matched unipolar depressed and healthy subjects. To our knowledge, this is the only neuroimaging study comparing treatment-naïve patients with bipolar II depression and unipolar depression.

Our findings stand in contrast to existing reports of widespread WM loss that were summarised in a recent ENIGMA meta-analysis of DTI studies on 1482 BD patients, which reported widespread WM abnormalities in BD, with no significant difference between the two subtypes^9,18^. This discrepancy could ensue from a few salient features in our sample.

Firstly, before we disregard the potential impact of WM in the bipolar subtypes, we need to consider firstly, that the lack of a BPI group precluded direct comparison with BPII subjects in our study, while two previous studies did report relative sparing of WM changes in uncinate fasciculus and corpus callosum in BPII compared to BPI and unaffected siblings^9,14^, which is consistent with the absence of WM loss in this BPII sample, and our earlier report of relatively preserved cognitive functioning of young treatment-naïve BPII patients^21^. These three studies all suffered from relatively small sample sizes (32, 58 and 20 BD, respectively), Further studies including probands and unaffected siblings of both BPI and BPII for comparison would be needed.

Secondly, our sample was medication-naïve. It is premature to conclude if the absence of WM disruption in our BPII sample was attributable to medication naivety. To begin with, structural and functional changes have been shown to commence in BD before receiving any medication^22,23^, and continued to be seen after medications were stopped for > 2 months^24,25^. On the other hand, studies on the effect of medications on brain structure in BD are conflicting. An earlier review suggested that medications generally did not contribute to structural differences between BD and HC, and where the effect was present, it was usually normalizing^26^. However, the authors also noted that many of the comparisons between medicated and unmedicated subjects were underpowered (sample sizes as small as n = 2), and a recent meta-analysis identified reduced FA in patients receiving antipsychotics and anticonvulsants in multiple regions of interest^18^. Multiple lines of evidences support the neuroprotective effects of mood stabilizers and lithium^18,26–28^, although one study did observe reduced FA only in lithium-treated but not lithium-free BD versus HC^29^. Clarifying the effects of different drugs in medicated samples requires overcoming numerous challenges in sample size limitations, variations in medication load, combinations and adherence, the common occurrence of polypharmacy managing bipolarity, and also complex symptomatic differences that influence choice of medication use.

Unfortunately, examination of unmedicated samples have been rare. One study of unmedicated (eight of the 18 patients were treatment-naïve) pediatric BP-I patients did not find any WM disruptions^30^, but reduced FA in superior frontal regions was reported in a small sample of medication-naïve adolescent BP-I patients in their first manic episode (n = 11)^31^. While medications are not expected to entirely explain the heterogeneity of the WM findings as noted above, examination of treatment-naïve samples should allow better comparability and removal of its confounding effects especially in the comparison of structural connectivity measures between bipolar and unipolar samples.

Alternatively, the paucity of white matter disruption in these young (average age 23), treatment-naïve bipolar II patients with an average of 5 years of illness may suggest that the wide spread WM disruptions summarised in the ENIGMA meta-analysis, with average age of 39.6 and on average 15.47 years since illness onset^9,18^, could be attributable to neuroprogression. Neuroprogression in bipolar disorder refers to the accumulation of biological disruptions, such as increase in pro-inflammatory cytokines and reduction in neurotrophins, following acute mood instability which progressively increase patients’ vulnerability to subsequent affective episodes^32^. This is consistent with previously reported correlations of illness chronicity with impaired WM integrity^6,33^, although WM disruptions had also been found in samples with shorter illness durations (0.2–5.6 years)^9^, individuals at high familial risk of BD who have not experienced any affective episodes^6^, and changes in brain volume and functional connectivity have been shown in first-episode BD patients^22,34^. Late onset and short disease duration were also found in the ENIGMA meta-analysis to be correlated with higher FA in multiple ROIs^18^. In fact, our observation of significant group-by-age interaction in MD, with age affecting MD in the right ILF specifically in BPII (but not UD and HC) appears consistent with existing evidence of neuroprogression leading to WM damage as a specific phenomenon in bipolar disorder.

The significant reduction in UD patients, compared to BPII and HC, of AD in the right SLF, may reflect increased WM disruption^35^ in medication-naïve UD but not individuals with BPII. Decreased AD has previously been reported in first-episode treatment-naïve UD patients, but in left rather than right SLF^36^. The lack of between-group difference in RD with reduced axial diffusivity suggested that impaired axonal integrity, instead of myelin loss, explained the reduction of MD, which is a scalar estimate of mean water diffusivity perpendicular and parallel to the tract^37^. The difference was unlikely related to clinical severity or chronicity effects, as UD and BPII groups had similar depressive and anxiety severity scores, number of comorbid disorders and significantly fewer affective episodes in UD. The two groups’ similar percentages of patients with family depressive history also did not suggest stronger biological loading in the UD group.

The negative correlation between family history of MDD and AD in the right SLF UD group may suggest a link of heritable susceptibility to depressive disorder to impaired axonal integrity in UD. Indeed, altered WM integrity in multiple ROIs including bilateral SLF, has been shown in healthy adolescents at familial risk for affective disorders^38^. Interestingly, in the BPII group, AD in right SLF correlated negatively with bipolarity index, suggesting a potential relationship between inherent and manifest bipolarity and axonal damage, but given the multidimensional nature of this index^39^, it would require larger samples to elucidate whether it was the family bipolarity, course or symptom characteristics that explained the correlation. The positive correlation of family history of MDD with AD in right SLF in the BPII group is in conflict with the negative correlation observed in the UD sample. Although consistent with our ANOVA result that BPII group has increased AD in right SLF, this finding suggests that familial risk of depressive disorder may relate to increased axonal integrity in patients with bipolar disorder. This finding needs to be further examined in larger samples, which could benefit from combined analysis of comparable samples in multi-centre studies.

WM disruption in the SLF had been reported in medication-free patients with UD^40^ to be associated with depressive symptoms and a possible trait marker for late-life depression. For right SLF (temporal) integrity (AD) to co-vary only with trait (family bipolarity) and symptom (hypomanic) markers of bipolarity in the PCA results in the present study, and to discriminate UD from BPII with good sensitivity and specificity in LDA among the depressed patients, along with a separate component that indicates episodic recurrence (known marker for bipolarity), in fact suggested right SLF (temporal) integrity to be a potential discriminant marker for bipolar II vs unipolar depression that is independent of course characteristics. However, the sensitivity and specificity in this exploratory LDA was likely inflated by the application of this model on the same samples from which the measures were chosen based on one-way ANOVAs to differentiate the two samples. To compensate for this shortcoming, we performed another LDA with leave-one-out cross-validation and found similar prediction accuracies (see Supplementary Table S3), which ensured that the accuracy of the selected training sample for deriving discriminant scores was optimistic. Nonetheless, these factors should be further tested on samples recruited de novo, which would greatly benefit from multi-centre collaboration.

There are other limitations that need to be considered in interpreting the findings here. Firstly, WM integrity is only one of many factors affecting DTI indices^41^. Even though the 15 a priori WM tracts are well-characterised^42^, there are still likely crossing fibres present in some of the tracts to affect water diffusion. Any such group differences in tissue fibre architecture, as well as axon diameter and packing density are all potential confounds that could have obscured WM disruptions in BD. Secondly, our sample may have excluded more severely depressed or suicidal patients, who would have required immediate inpatient treatment, which may render brain scan in a treatment-naïve state risky and ethically questionable. To the extent that these such patients may be expected to have greater biological disturbances, our findings may have underestimated WM disruptions in BD and UD. Thirdly, a larger sample size, or meta-analyses may enhance power that is needed to detect smaller white matter changes and examine the effects of clinical bipolar correlates of possibly smaller effect size, such as family loading and age of onset. Fourth, the lack of a BPI comparison group precludes direct comparison with our BPII patients. As mentioned, it is therefore uncertain if the observed differences (or lack thereof) between UD and BPII were specific to BPII or applicable to BPI as well. Fifth, the exclusion of psychosis or substance use has allowed us to conduct a more homogeneous investigation, but may have also limited us from accessing a fuller spectrum of bipolarity which would be associated with these two conditions. Lastly, we do not have data on the subjects’ body mass index, which were reported to be associated with WM abnormalities^43^. Although none of our young and medication-naïve patients reported metabolic syndromes, general medical illnesses frequently comorbid with BD such as obesity, hypertension and diabetes^44^, may confound the WM disruptions observed in DTI studies and should be included in further research.

The imaging and DTI processing approaches adopted in our study may have shortcomings compared to those used in the Human Connectome Project (HCP). The global probabilistic tractography used in this study (TRACULA) enabled fast and automated quantification of WM tracts in individual subject’s space, eliminating the inaccuracies caused by inter-subject registration. However, with this approach, a single FA (and other DTI indices) was used to quantify the diffusion asymmetry of the entire tract, which might have diluted any WM abnormalities that were not present universally along the tract. Also, tractography was performed for the 15 WM tracts pre-defined by the toolbox only, which may overlook the possibility of other tracts and brain areas that relate to the psychopathological conditions of interest. Alternatively, TBSS enabled voxel-wise comparison of WM tracts between different groups of participants, but reliability has been proven to depend heavily on the specific DTI-derived measure and the pre-processing steps (e.g. warping subjects to a common individual template, smoothing DTI data, etc.)^45^. On the one hand, these methods allow automated and straightforward processing of DTI data, which is best suited for exploratory analyses. On the other hand, more advanced imaging and processing approaches are useful for characterizing the structural, as well as functional, connectivity in bipolar II disorder holistically. Indeed, the state-of-the-art HCP has enabled advances in imaging and processing pipelines that improve efficiency and accuracy of MRI data analysis^46^. For example, the ‘multi-band’ pulse sequences^47^ and customized scanners with increased maximum gradient strength benefit diffusion MRI by enhancing spatial resolution (1.25 mm)^48^, which facilitates identification of cross-fibers. Scanners with increased diffusion gradient directions have also been developed to improve sensitivity of tractography and support more sophisticated models^49,50^. An increasing body of research has used probabilistic tractography to build connection matrix that summarizes the macroscopic connectivity of *every* brain area. In addition, whole brain connectomics analysis of this dataset is under way, which is hoped to shed light on differences in the network characteristics of these BPII and UD patients. Further efforts will also benefit from alignment with the state-of-the-art HCP pipelines in image acquisition and pre-processing.

In summary, in this first DTI study comparing young and treatment-naïve BPII and UD patients, we could not find evidence of increased WM disruption in BPII disorder. Whether this represents a contemporaneous subtype difference between BPI and BPII merits direct, treatment-naïve, comparison. The discrepancy of our findings from previous BPII studies also belies the methodological salience in examining patients stratified by medication status and illness chronicity. Nonetheless, that we could not find evidence of WM disruption in medication-naïve young adults with on average 5 years of illness history suggest that the WM disruptions otherwise found in more chronically ill bipolar patients may be substantially attributable to effects of illness chronicity. The findings also encourage longitudinal examination for the specific effects of accumulated illness and recurrence in BPII disorder, which would help establish the biological underpinning of bipolarity to the extent that it is observable versus BPI and UD.

## Methods

### Participants and recruitment

Treatment-naïve, currently depressed subjects were recruited from individuals presenting to either of three specialist psychiatric clinics during the years 2014–2018 for scheduling a new appointment. Inclusion criteria were (1) aged 18–30, (2) currently satisfying the criteria for DSM-IV-TR Major Depressive Episode, (3) either meeting DSM-IV-TR criteria for Major Depressive Disorder, with no history of hypomanic episodes (for UD), or meeting research diagnostic criteria (RDC) for Bipolar II Disorder (BPII) (DSM-IV-TR MDE with history of hypomanic episodes of at least 2-day duration)^51^, and (4) no prior exposure to any psychotropic drug treatment in their life time. Exclusion criteria included: (1) current and lifetime histories of psychoses, (2) substance misuse, (3) organic brain syndromes, and/or (4) evidence of intellectual disability. Healthy volunteers without personal or family history of any mental disorders were recruited from online advertisements and a public health centre.

Written informed consent was obtained from all subjects/patients. The authors assert that all procedures contributing to this work comply with the ethical standards of the relevant national and institutional committees on human experimentation and with the Helsinki Declaration of 1975, as revised in 2008. All procedures involving human subjects/patients were approved by the New Territories East Cluster—Chinese University of Hong Kong Clinical Research Ethics Committee (CREC Ref. No.: 2014.168).

### Clinical assessments

Diagnostic assessments were conducted by trained interviewers using the Chinese bilingual version of the Structured Clinical Interview for DSM-IV Axis I Disorders (SCID-I), adapted to facilitate diagnosis of current and lifetime hypomanic episodes under the supervision of an experienced clinician academic psychiatrist^51–53^. All lifetime affective episodes were enquired year by year from the first onset of depression using a modified life-chart method based on SCID-I. Repeated interviews were conducted to enhance detection of past hypomanic episodes.

Current-week affective symptoms were rated by trained clinician interviewers using the interviewer-administered MADRS^54^, YMRS^55^, and HAM-A^56^. Current health-related quality of life was evaluated with the SF-36^57^, validated for Chinese settings^58^. Medication history was directly enquired with participants and caregivers, and checked against the territory-wide public hospital computer registry. We also assessed for the Bipolarity Index, a clinician-rated scale across five domains: signs and symptoms, age of onset, course of illness, responses to treatment and family history denoting bipolarity as a dimensional construct^39^. General intelligence was measured with the three-subtest short form of Wechsler Adult Intelligence Scale-III (WAIS-III)^59,60^.

### MRI acquisition

MR images were acquired using 3-T MRI scanner (Achieva TX series, Philips Healthcare, Best, Netherlands) using a standard 8-channel head coil for signal reception. High resolution structural images of the whole brain were acquired using a 3D T1-weighted sequence (TR: 7.4 ms, TE: 3.4 ms, field of view: 250′250 mm^2^, 285 contiguous slices, sagittal plane, 0.6 mm R-L thickness, reconstruction matrix: 240′240, flip angle: 8°). To minimize the effects of patient motion to the quality of our raw data, padding was applied around to the participant's head to minimize motion during signal acquisition. At the end of each imaging sequence, image quality checks were made to ensure no gross motion artifacts were visible on the images.

Diffusion-weighted imaging was performed using a single-shot echo planar imaging sequence (TR: 8912 ms, TE: 60 ms, field of view = 224′224 mm^2^, 70 continuous axial slices, 2 mm slice thickness, no gap, acquisition matrix = 112′112, flip angle: 90°). Diffusion sensitizing gradients were applied along 32 non-linear directions with b = 1000 s/mm^2^, together with an acquisition without diffusion weighting (b = 0 s/mm^2^). A parallel imaging acceleration factor of 2.5 was used to reduce scan time.

### Image pre-processing

Image registration was performed to account for motion artifacts arising from patient motion and to realign image datasets before further post-processing. The anatomical images and diffusion weighted images were pre-processed using FSL^61^ and FreeSurfer 6.0^62^. The anatomical images were corrected for intensity non-uniformity^63^ in pursuance of segmentation. After removing the skull and neck, the brain images were registered to MNI-305 template, with a series of linear and non-linear transformations, for tract fitting. The brain surfaces were also constructed from brain images in individual native space.

The diffusion-weighted images (DWI) were pre-processed using Freesurfer, with the *trac-all* command. The DWI were first registered to the b0 images of the volume via affine transformation to correct for eddy-current and echo-planar imaging distortion and head motion, where the b-vectors were rotated to account for motion corrections. Individual DWI were then rigidly registered to individual anatomical images with aid of brain surface reconstruction cost function from anatomical image pre-processing. Cortical and white-matter masks were created, followed by tensor fitting for extraction of tensor-based measures (FA, MD, RD and AD). Lastly, the anatomical priors for 15 white-matter pathways (see below) were computed for TRACULA.

### TRACULA

TRACULA (TRActs Constrained by UnderLying Anatomy)^42^, an automated toolbox within Freesurfer, was applied to reconstruct the global probabilistic distribution of 15 a priori, well-characterized white matter tracts implicated in bipolar disorder^18,20^, including the anterior thalamic radiations (ATR), the cingulum cingulate gyrus (CING), the cingulum angular bundle (CAB), the inferior longitudinal fasciculus (ILF), the parietal part of the SLF, the temporal part of the SLF, the uncinate fasciculus (UNC), *all bilaterally*; and the forceps minor of the corpus callosum (CC). To assure validity of findings, we also included three tracts with no reported bipolar association as control, namely forceps major and *bilateral* corticospinal tracts. Specifically, diffusion distributions were estimated by applying the ball-and-stick model, using “bedpostX” of FSL, followed by fitting the shape of each white-matter pathway to the results of the ball-and-stick model of diffusion and the anatomical priors for white-matter pathways computed during the pre-processing stage. Subsequently, we examined DTI-derived connectivity indices, including FA, MD, RD and AD (see below).

### TBSS

Tract-based spatial statistics (TBSS) was conducted using FSL for voxel-wise analysis of white matter tracts. FA, AD and RD images created using Freesurfer’s *trac-all* (see above) were projected onto the mean FA skeleton that represents the centre of white-matter tracts, thresholded at FA = 0.2.

### Statistical analysis

Statistical tests for demographic, clinical and imaging (TRACULA) data were performed using R Statistical Software^64^. Group differences of demographic and cognitive variables were analysed using one-way ANOVA for continuous variables and Chi-Squared test for categorical variables. Post-hoc group comparisons were performed with false discovery rate (FDR) correction. Differences of clinical measures between UD and BPII patients were analysed by means of independent t-tests for continuous variables, and Chi-Square test for categorical variables. Where unequal variances existed between groups, Welch’s tests were used.

Group differences of FA, MD, RD and AD of unilateral tracts were analysed using one-way ANOVA, with WAIS-III IQ scores included in the model as covariate. In view of the small sample size and unequal variances of DTI data across groups, we performed data resampling by bootstrapping with 1000 iterations. With each bootstrap sample, we conducted a one-way ANOVA and extracted the bootstrapped ${F}_{B}$. For each DTI measure at each tract, we identified the 2.5th and 97.5th percentiles (i.e. 95% confidence intervals) of all ${F}_{B}$ and compared it with the observed F. Group differences were considered statistically significant where the observed F was not within the 95% CIs of ${F}_{B}$. Post-hoc t-tests were conducted with FDR correction. Using the same bootstrap samples (with 1000 iterations), the bootstrapped ${t}_{B}$ were extracted and the 2.5th and 97.5th percentiles (i.e. the 95% confidence intervals) of all ${t}_{B}$ were obtained. Significant group differences were identified where the observed t exceeded the 95% CIs of ${t}_{B}$. In addition to the main ANOVA, we conducted a supplementary group-by-age analysis to examine the effect of age on the DTI measures using the same ANOVA and bootstrapping methods. Results are reported in Supplementary Table S2. For TBSS, voxel-wise permutation analysis on the skeletonized data (FA, AD and RD) were performed using FSL’s PALM with 5000 permutations, using threshold-free cluster enhancement (TFCE), corrected for age, sex and education level.

We examined the correlations between the DTI and clinical data in our patient samples by calculating the Pearson’s *r*. Correlation was tested for each patient group separately and also with both groups combined. For all correlation tests, bootstrapping was conducted with 1000 iterations. Specifically, with each bootstrap sample, we extracted the ${r}_{B}$ and identified the 95% confidence intervals, which equalled to the 2.5th and 97.5th percentiles of all ${r}_{B}$. Significant correlation was indicated by which the observed *r* was not within the 95% CIs of ${r}_{B}$.

In view of multicollinearity in the clinical and imaging measures, principal components analysis (PCA) was performed, instead of linear regression, to assess the multivariate linear relationships among the clinical and DTI measures in the UD and BPII groups, on DTI measures and clinical variables with significant group differences. Varimax rotation was applied to the component loadings. We examined the scree plot and extracted components with eigenvalue greater than 1 to identify the number of components sufficient to explain the variance in the data. The components obtained from the PCA were then entered into a linear discriminant analysis (LDA) to examine the classification between UD and BPII groups based on the DTI and clinical variables. A maximum likelihood method was used, assuming the variables were continuous and normally distributed. To compensate for the relatively small sample size, we performed an LDA with leave-one-out cross-validation and found similar prediction accuracies (see Supplementary Table S3), which ensured that the accuracy of the selected training sample for deriving discriminant scores was optimistic. Since only DTI and clinical measures differentiating our UD and BPII samples were included in the PCA-LDA, the specificity and sensitivity in differentiating UD from BPII here is likely stronger here than would be found in the population. Thus, this analysis is exploratory in nature. In addition, PCA and LDA were performed again to examine the model without the DTI variable, i.e. AD in right SLF (temporal) to explore the effect of the imaging variable on the model.

## Supplementary Information

Supplementary Information.

## Data Availability

The datasets analysed during the current study are available from the corresponding author on reasonable request.

## References

[1] Bauer M, Pfennig A (2005). Epidemiology of bipolar disorders. Epilepsia.

[2] Dervic K (2015). Bipolar I and II versus unipolar depression: Clinical differences and impulsivity/aggression traits. Eur. Psychiatry.

[3] Sole B (2016). Cognitive variability in bipolar II disorder: Who is cognitively impaired and who is preserved. Bipolar Disord..

[4] Ghaemi, S. N. *et al.* Antidepressant treatment in bipolar versus unipolar depression. *Am. J. Psychiatry* **161**, 163–165. 10.1176/appi.ajp.161.1.163 (2004). 10.1176/appi.ajp.161.1.163 14702267

[5] Tondo L, Lepri B, Baldessarini RJ (2007). Suicidal risks among 2826 Sardinian major affective disorder patients. Acta Psychiatr. Scand..

[6] Sprooten E (2013). Reduced white matter integrity in sibling pairs discordant for bipolar disorder. Am. J. Psychiatry.

[7] Benedetti F (2011). Tract-specific white matter structural disruption in patients with bipolar disorder. Bipolar Disord..

[8] McIntosh AM (2008). White matter tractography in bipolar disorder and schizophrenia. Biol. Psychiatry.

[9] Lagopoulos J (2013). Microstructural white matter changes in the corpus callosum of young people with Bipolar Disorder: A diffusion tensor imaging study. PLoS ONE.

[10] Versace A (2010). Right orbitofrontal corticolimbic and left corticocortical white matter connectivity differentiate bipolar and unipolar depression. Biol. Psychiatry.

[11] Han KM, De Berardis D, Fornaro M, Kim YK (2019). Differentiating between bipolar and unipolar depression in functional and structural MRI studies. Prog. Neuropsychopharmacol. Biol. Psychiatry.

[12] Repple J (2017). A voxel-based diffusion tensor imaging study in unipolar and bipolar depression. Bipolar Disord..

[13] Liu JX (2010). Differences in white matter abnormalities between bipolar I and II disorders. J. Affect Disord..

[14] Foley SF (2018). Fractional anisotropy of the uncinate fasciculus and cingulum in bipolar disorder type I, type II, unaffected siblings and healthy controls. Br. J. Psychiatry.

[15] Borgwardt S, Fusar-Poli P (2012). White matter pathology—An endophenotype for bipolar disorder?. BMC Psychiatry.

[16] Berk M (2011). Pathways underlying neuroprogression in bipolar disorder: Focus on inflammation, oxidative stress and neurotrophic factors. Neurosci. Biobehav. Rev..

[17] Abramovic L (2018). White matter disruptions in patients with bipolar disorder. Eur. Neuropsychopharmacol..

[18] Favre P (2019). Widespread white matter microstructural abnormalities in bipolar disorder: Evidence from mega- and meta-analyses across 3033 individuals. Neuropsychopharmacology.

[19] Mamah D, Ji A, Rutlin J, Shimony JS (2019). White matter integrity in schizophrenia and bipolar disorder: Tract- and voxel-based analyses of diffusion data from the Connectom scanner. Neuroimage Clin..

[20] Versace A (2014). Elevated serum measures of lipid peroxidation and abnormal prefrontal white matter in euthymic bipolar adults: Toward peripheral biomarkers of bipolar disorder. Mol. Psychiatry.

[21] Mak ADP (2018). Cognitive impairment in treatment-naive bipolar II and unipolar depression. Sci. Rep..

[22] Atmaca M, Ozdemir H, Yildirim H (2007). Corpus callosum areas in first-episode patients with bipolar disorder. Psychol. Med..

[23] Jiang X (2021). Structural and functional alterations in untreated patients with major depressive disorder and bipolar disorder experiencing first depressive episode: A magnetic resonance imaging study combined with follow-up. J. Affect Disord..

[24] Savitz JB (2011). Habenula volume in bipolar disorder and major depressive disorder: A high-resolution magnetic resonance imaging study. Biol. Psychiatry.

[25] Rive MM (2016). Distinguishing medication-free subjects with unipolar disorder from subjects with bipolar disorder: state matters. Bipolar Disord..

[26] Hafeman DM, Chang KD, Garrett AS, Sanders EM, Phillips ML (2012). Effects of medication on neuroimaging findings in bipolar disorder: An updated review. Bipolar Disord..

[27] Versace A (2008). Elevated left and reduced right orbitomedial prefrontal fractional anisotropy in adults with bipolar disorder revealed by tract-based spatial statistics. Arch. Gen. Psychiatry.

[28] Atmaca M, Yildirim H, Ozdemir H, Ogur E, Tezcan E (2007). Hippocampal 1H MRS in patients with bipolar disorder taking valproate versus valproate plus quetiapine. Psychol. Med..

[29] Benedetti F (2011). Disruption of white matter integrity in bipolar depression as a possible structural marker of illness. Biol. Psychiatry.

[30] Teixeira AM (2014). Preserved white matter in unmedicated pediatric bipolar disorder. Neurosci. Lett..

[31] Adler CM (2006). Evidence of white matter pathology in bipolar disorder adolescents experiencing their first episode of mania: A diffusion tensor imaging study. Am. J. Psychiatry.

[32] Grande I, Magalhaes PV, Kunz M, Vieta E, Kapczinski F (2012). Mediators of allostasis and systemic toxicity in bipolar disorder. Physiol. Behav..

[33] Zanetti MV (2009). State-dependent microstructural white matter changes in bipolar I depression. Eur. Arch. Psychiatry Clin. Neurosci..

[34] Yin Z (2018). Decreased functional connectivity in insular subregions in depressive episodes of bipolar disorder and major depressive disorder. Front. Neurosci..

[35] Aung WY, Mar S, Benzinger TL (2013). Diffusion tensor MRI as a biomarker in axonal and myelin damage. Imaging Med..

[36] Lai CH, Wu YT (2014). Alterations in white matter micro-integrity of the superior longitudinal fasciculus and anterior thalamic radiation of young adult patients with depression. Psychol. Med..

[37] Winklewski PJ (2018). Understanding the physiopathology behind axial and radial diffusivity changes—What do we know?. Front. Neurol..

[38] Huang, H., Fan, X. Williamson, D. E., & Rao, U. White matter changes in healthy adolescents at familial risk for unipolar depression: A diffusion tensor imaging study. *Neuropsychopharmacology* **36**, 684–691. 10.1038/npp.2010.199 (2011). 10.1038/npp.2010.199 PMC3036948 21085111

[39] Aiken CB, Weisler RH, Sachs GS (2015). The Bipolarity Index: A clinician-rated measure of diagnostic confidence. J. Affect. Disord..

[40] Jiang J (2017). Microstructural brain abnormalities in medication-free patients with major depressive disorder: A systematic review and meta-analysis of diffusion tensor imaging. J. Psychiatry Neurosci. JPN.

[41] Jones DK, Knosche TR, Turner R (2013). White matter integrity, fiber count, and other fallacies: The do's and don'ts of diffusion MRI. Neuroimage.

[42] Yendiki A, Panneck P, Srinivasan P, Stevens A, Zöllei L, Augustinack J, Wang R, Salat D, Ehrlich S, Behrens T, Jbabdi S, Gollub R, Fischl B (2011). Automated probabilistic reconstruction of white-matter pathways in health and disease using an atlas of the underlying anatomy. Front. Neuroinform..

[43] Gunde E, Blagdon R, Hajek T (2011). White matter hyperintensities: From medical comorbidities to bipolar disorders and back. Ann. Med..

[44] Fiedorowicz JG, Palagummi NM, Forman-Hoffman VL, Miller DD, Haynes WG (2008). Elevated prevalence of obesity, metabolic syndrome, and cardiovascular risk factors in bipolar disorder. Ann. Clin. Psychiatry.

[45] Madhyastha T, Mérillat S, Hirsiger S, Bezzola L, Liem F, Grabowski T, Jäncke L (2014). Longitudinal reliability of tract-based spatial statistics in diffusion tensor imaging. Hum. Brain Mapp..

[46] Van Essen DC (2013). The WU-Minn human connectome project: An overview. Neuroimage.

[47] Ugurbil K (2013). Pushing spatial and temporal resolution for functional and diffusion MRI in the Human Connectome Project. Neuroimage.

[48] Sotiropoulos SN (2013). Advances in diffusion MRI acquisition and processing in the Human Connectome Project. Neuroimage.

[49] Shi Y, Toga AW (2017). Connectome imaging for mapping human brain pathways. Mol. Psychiatry.

[50] Andersson JLR, Sotiropoulos SN (2015). Non-parametric representation and prediction of single- and multi-shell diffusion-weighted MRI data using Gaussian processes. NeuroImage.

[51] Benazzi F, Akiskal HS (2003). Refining the evaluation of bipolar II: Beyond the strict SCID-CV guidelines for hypomania. J. Affect. Disord..

[52] Mak ADP (2009). Prevalence and correlates of bipolar II disorder in major depressive patients at a psychiatric outpatient clinic in Hong Kong. J. Affect. Disord..

[53] So E, Kam I, Leung CM, Chung D, Liu Z, Fong S (2003). The Chinese-bilingual SCID-I/P Project: Stage 1—Reliability for mood disorders and schizophrenia. Hong Kong J. Psychiatry.

[54] Montgomery, S. A., & Asberg, M. A new depression scale designed to be sensitive to change. *Br. J. Psychiatry J. Mental Sci.* **134**, 382–389. 10.1192/bjp.134.4.382 (1979). 10.1192/bjp.134.4.382 444788

[55] Young, R. C., Biggs, J. T., Zieger, V. E., & Meyer, D. A. A rating scale for mania: reliability, validity and sensitivity. *Br. J. Psychiatry J. Mental Sci.* **133**, 429–435. 10.1192/bjp.133.5.429 (1978). 10.1192/bjp.133.5.429 728692

[56] Hamilton M (1959). The assessment of anxiety states by rating. Br. J. Med. Psychol..

[57] Brazier JE, Harper R, Jones NM, O’Cathain A, Thomas KJ, Usherwood T, Westlake L (1992). Validating the SF-36 health survey questionnaire: New outcome measure for primary care. BMJ.

[58] Lam CL, Tse EY, Gandek B, Fong DY (2005). The SF-36 summary scales were valid, reliable, and equivalent in a Chinese population. J. Clin. Epidemiol..

[59] Wechsler D (1987). Wechsler Memory Scale-Revised Manual.

[60] Chan ELS, Chen EYH, Chan RCK (2005). Three-subtest short form of the wechsler adult intelligence scale-III for patients with psychotic disorders: A preliminary report. Hong Kong J. Psychiatry.

[61] Jenkinson M, Bechmann CF, Behrens TE, Woolrich MW, Smith SM (2012). FSL. Neuroimage.

[62] Reuter M, Schmansky NJ, Rosas HD, Fischl B (2012). Within-subject template estimation for unbiased longitudinal image analysis. Neuroimage.

[63] Zheng W, Chee MW, Zagorodnov V (2009). Improvement of brain segmentation accuracy by optimizing non-uniformity correction using N3. Neuroimage.

[64] R Core Team. R: A language and environment for statistical computing. R Foundation for Statistical Computing, Vienna, Austria. (2017).

