# Supplementary material for: White matter integrity in young medication-naïve bipolar II depressed adults
Source: Sci Rep. 2021 Jan 19;11:1816. doi: 10.1038/s41598-021-81355-9 (PMC7815920; doi:10.1038/s41598-021-81355-9)
Supplement: Supplementary file 1 — Supplementary Information. [file 41598_2021_81355_MOESM1_ESM.docx]

**White Matter Integrity in Young Medication-naïve Bipolar II Depressed Adults**

**Supplementary Information**

Mak, Arthur Dun Ping^1^

Leung, Owen Ngo Wang^1^

Chou, Idy Wing Yi^1^

Wong, Sheila Lok Yiu^1^

Chu, Winnie Chiu-wing^2^

Yeung, David^3^

So, Suzanne Ho-wai^4^

Ma, Suk Ling^1^

Lam, Linda Chiu Wah^1^

Leung, Chi Ming^1^

Lee, Sing^1^

| **Supplementary Table S1.** Demographic and clinical data | | | | | | | | | | | |
| --- | --- | --- | --- | --- | --- | --- | --- | --- | --- | --- | --- |
|  |  |  |  |  |  |  |  |  | Effect size | | |
| Variable | HC (n=27) | UD (n=27) | BPII (n=27) | *t* | *F* | *X^2^* | *DOF* | *p* | *Cohen's d* | ηp^2^ | *φc* |
| Gender, female^1^ | 17 (62.96) | 18 (66.67) | 21 (77.77) | - | - | 1.5 | 2 | 0.47 | - | - | 0.12 |
| Age^2^ | 22.70 ± 3.37 | 23.96 ± 3.96 | 23.07 ± 4.02 | - | 0.79 | - | 2, 78 | 0.46 | - | 0.02 | - |
| Years of education^1^ | | | | - | - | 5.12 | 4 | 0.28 | - | - | 0.13 |
| Pre-secondary | 0 (0) | 4 (14.81) | 4 (14.81) |  |  |  |  |  |  |  |  |
| Secondary | 4 (14.81) | 5 (18.52) | 3 (11.11) |  |  |  |  |  |  |  |  |
| Tertiary | 23 (85.19) | 18 (66.67) | 20 (74.07) |  |  |  |  |  |  |  |  |
| MADRS^2^ | - | 25.56 ± 5.24 | 26.15 ± 9.25 | 0.29 | - | - | 52 | 0.86 | 0.08 | - | - |
| YMRS^2^ | - | 1.33 ± 2.00 | 4.22 ± 4.24 | 3.20* | - | - | 52 | 0.02 | 0.87 | - | - |
| HAM-A^2^ | - | 19.81 ± 8.04 | 19.11 ± 7.66 | -0.33 | - | - | 52 | 0.86 | 0.09 | - | - |
| Year since depressive onset^2^ | - | 2.99 ± 3.33 | 4.96 ± 3.96 | 1.97 | - | - | 52 | 0.15 | 0.54 | - | - |
| Year since hypomanic onset^2^ | - | - | 2.79 ± 3.56 | - | - | - | - | - | - | - | - |
| Total number of MDE^2^ | - | 1.44 ± 0.75 | 2.37 ± 1.23 | 2.41* | - | - | 52 | 0.02 | 0.91 | - | - |
| Total number of HME^2^ | - | - | 48.41 ± 95.11 | - | - | - | 52 | - |  | - | - |
| Number of episodes in the past year^2^ | - | 1.11 ± 0.32 | 9.93 ± 16.29 | 2.81* | - | - | 52 | 0.04 | 0.77 | - | - |
| Lifetime total number of episodes^2^ |  | 1.70 ± 1.03 | 50.85 ± 95.47 | 2.64* | - | - | 52 | 0.04 | 0.73 | - | - |
| Rapid cycling specifier^1^ | - | - | 9 (33.33) | - | - | - | - | - | - | - | - |
| Bipolarity Index^2^ | - | 19.70 ± 6.89 | 35.22 ± 10.23 | 6.54*** | - | - | 52 | < .001 | 1.78 | - | - |
| Lifetime number of comorbid disorders^2^ | - | 2.11 ± 1.45 | 1.67 ± 1.21 | -1.22 | - | - | 52 | 0.48 | 0.33 | - | - |
| Lifetime number of anxiety disorders^2^ | - | 1.70 ± 0.91 | 1.44 ± 1.12 | -0.93 | - | - | 52 | 0.68 | 0.25 | - | - |
| SF-36 PCS^2^ | - | 51.48 ± 7.03 | 51.08 ± 7.54 | -0.2 | - | - | 52 | 0.89 | 0.05 | - | - |
| SF-36 MCS^2^ | - | 24.28 ± 8.62 | 26.08 ± 10.84 | 0.68 | - | - | 52 | 0.73 | 0.18 | - | - |
| Family history of MDD^1^ | - | 20 (74.07) | 22 (81.48) | - | - | 0.11 | 1 | 0.74 | - | - | 0.05 |
| Family history of HM^1^ | - | 2 (7.41) | 11 (40.74) | - | - | 6.48* | 1 | 0.01 | - | - | 0.28 |
| WAIS-III IQ estimate^2^ | 35.22 ± 7.49 | 29.11 ± 7.83 | 30.77 ± 6.45^#^ | - | 5.07** | - | 2, 77 | 0.009 |  | 0.12 | - |
| HC, Healthy control; UD, Unipolar Depression; BPII, Bipolar II Disorder; DOF, degrees of freedom; MADRS, Montgomery–Åsberg Depression Rating Scale; YMRS, Young Mania Rating Scale; HAM-A, Hamilton Anxiety Rating Scale; MDE, Major Depressive Episode; HME, Hypomanic Episode; SF-36, 36-Item Short Form Survey; PCS, Physical component summary; MCS, Mental component summary; WAIS-III IQ, Wechsler Adult Intelligence Scale-III.  ^1^ n(%)  ^2^ mean(SD)  ^#^ n = 26 | | | | | | | | | | | |

| **Supplementary Table S2. Results of group-by-age ANOVA on DTI measures in right inferior longitudinal fasciculus** | | | | | | | | | | | | | | | | |  |  |
| --- | --- | --- | --- | --- | --- | --- | --- | --- | --- | --- | --- | --- | --- | --- | --- | --- | --- | --- |
| *Measure* | | | *UD (N = 27)* | | *BPII (N = 27)* | | *HC (N = 27)* | | *DOF* | | *F* | | | *95% CI of* $F_{B}$ | | |  |  |
|  |  |  |  |  |  |  |  |  |  |  |  |  |  |  |  |  |  | |
| MD | | | .77^ ± .02^ | | .78^ ± .02^ | | .77^ ± .02^ | | 2, 78 | | 5.28 | | | .001, 5.02 | | |  | |
| Post-hoc one-way ANOVA (effect of age on MD) | | | | | | | | | | | | | | | | |  | |
|  | *UD* | | | | | *BPII* | | | | | | | *HC* | | | |  | |
| *DOF* | | *F* | | *95% CI of* $F_{B}$ | | *DOF* | | *F* | | *95% CI of* $F_{B}$ | | *DOF* | | | *F* | *95% CI of* $F_{B}$ |  |  |
| 26 | | .77^ | | .001, 5.84 | | 26 | | 19.51* | | .001, 5.31 | | 26 | | | .008 | .001, 5.57 |  |  |
| ^^^Multiply by 10^-3^  *Statistically significant, where the observed statistics was not within the 95% CIs of the bootstrapped statistics  $F_{B}$: bootstrapped F statistics; DOF: degrees of freedom; CI: Confidence intervals; UD: Unipolar Depression; BPII: Bipolar II Disorder; HC: Healthy control; MD: mean diffusivity | | | | | | | | | | | | | | | | |  | |

| **Supplementary Table S3. Prediction accuracies of LDA with and without leave-one-out cross-validation** | | | | | |
| --- | --- | --- | --- | --- | --- |
| LDA with cross-validation | | | LDA without cross-validation | | |
|  | Predicted |  |  | Predicted |  |
| Actual | *UD* | *BPII* | Actual | *UD* | *BPII* |
| *UD* | .889 | .111 | *UD* | .926 | .074 |
| *BPII* | .370 | .630 | *BPII* | .370 | .630 |

LDA: linear discriminant analysis; UD: unipolar depression; BPII: bipolar II disorder

**Supplementary Figure S1. PCA and LDA results with clinical variables showing between-group difference**


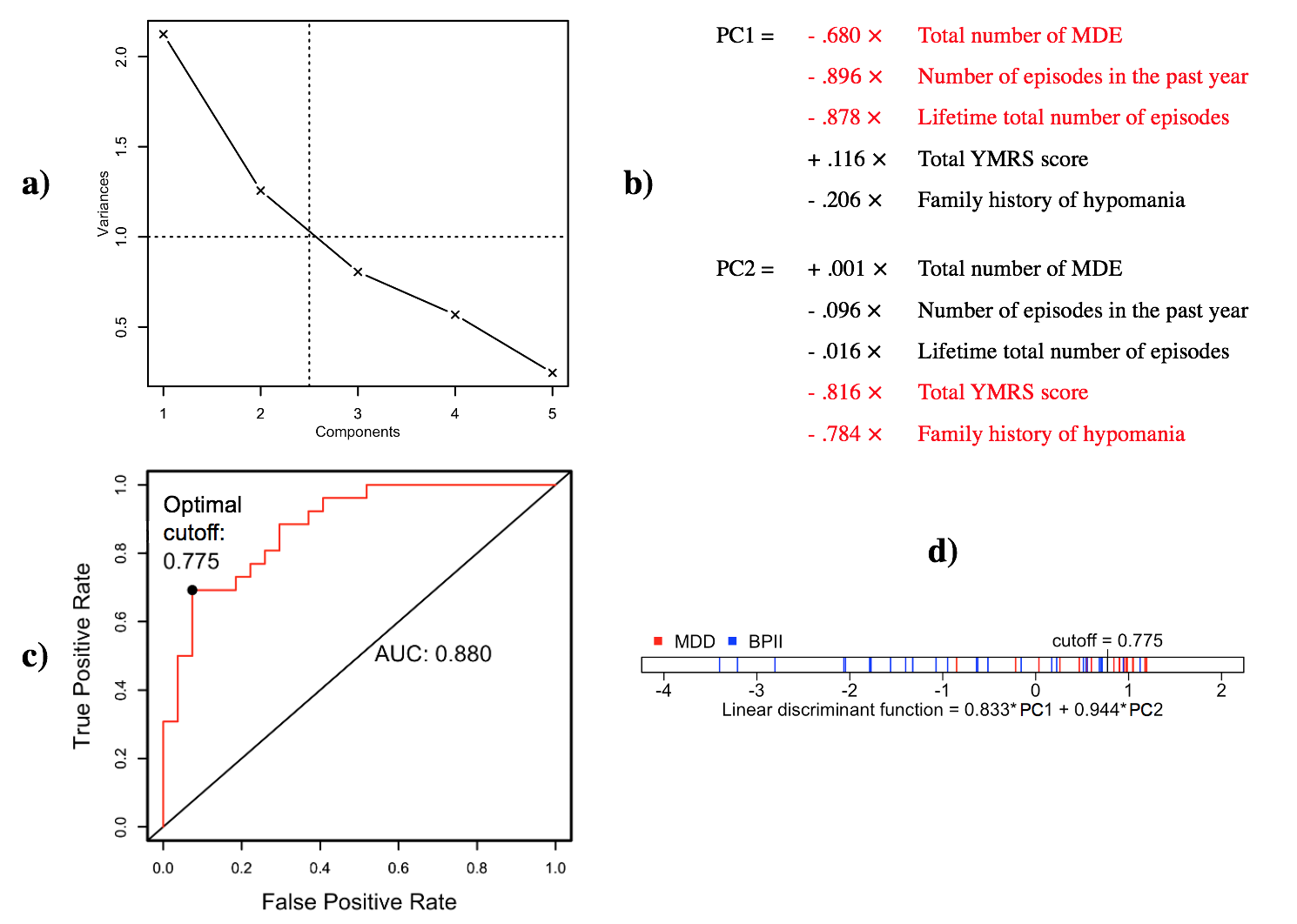


Figure 2 a) Scree plots showing the amount of variance explained by each component in the principal component analysis b) Principal component analysis loadings c) Receiver operating curve of the function d) Linear Discriminant Function
